# Tri‑, Tetra‑, Octa-Nuclear Copper Complexes Including the First Mode Cubane-like {Cu_4_O_3_N} Core: Synthesis, Structure, and Magnetic Properties

**DOI:** 10.1021/acsomega.5c11893

**Published:** 2026-06-18

**Authors:** Cándida Pastor-Ramírez, Sylvain Bernès, Rafael Zamorano-Ulloa, Daniel Ramírez-Rosales, Samuel Hernández-Anzaldo, Yasmi Reyes-Ortega

**Affiliations:** † 3972 Benemérita Universidad Autónoma de Puebla, Instituto de Ciencias Químicas BUAP, Av. San Claudio y 24 sur S/N, Col San Manuel, C. P., 72570 Puebla, Puebla, Mexico; ‡ Benemérita Universidad Autónoma de Puebla, Instituto de Física IFUAP, Av. San Claudio y 18 sur S/N, Col San Manuel, C. P., 72570 Puebla, Puebla, Mexico; § Instituto Politécnico Nacional-Escuela Superior de Física y Matemáticas-IPN, Avenida Instituto Politécnico Nacional S/N, Gustavo A. Madero, San Pedro Zacatenco, C. P., 07738 Ciudad de México, Mexico

## Abstract

In this study, the synthesis and characterization of three Cu­(II) complexes employing the Schiff base ligand 2-[(2-hydroxy-benzylidene)-amino]-2-hydroxymethylpropane-1,3-diol (H_4_L), sodium azide (NaN_3_), and Cu­(II) salts were investigated. This research led to the formation of [Cu_4_(H_2_L)_4_(H_2_O)] **1**, previously reported, the first octa-nuclear cubane-like compound based on the {Cu_4_O_3_N} core, [Cu_4_(μ_3_-N_3_)­(H_2_L)_2_(HL)­(H_2_O)]_2_ **2**, and a polymeric compound [Cu_3_(H_2_L)_2_(μ_2_-N_3_)_2_]_n_ **3**. Single-crystal X-ray diffraction analysis revealed that **1·**CH_3_OH possesses a ‘4 + 2’ cubane-type core, **2** is a double cubane of eight Cu­(II) ions including μ_3_-alkoxido and μ_3_-azido bridges, while **3** is a polymeric structure with three Cu­(II) ions conforming the monomeric unit. Temperature-dependent (2.9–300 K) magnetic susceptibility measurements demonstrate the coexistence of ferromagnetic and antiferromagnetic spin exchange interactions in **1**, associated with Cu–O–Cu bridging angles ranging from 77.81(6)° to 112.77(8)°. Compounds **2** and **3** exhibited antiferromagnetic exchange coupling, attributed to their large Cu–O–Cu bond angles, the largest of which was 108.92°. The ESR spectra of **1**–**3** at 300 and 90 K are axial, accompanied by hyperfine splitting in *g*_∥_ and coupling constants *A*_Cu_ in the range 169 × 10^–4^ cm^–1^ to 197 × 10^–4^ cm^–1^. In accordance with *g*_⊥_ values >2.0023, the unpaired electron ground state is $d_{x^{2}-y^{2}}$ , consistent with a Cu­(II) ion. Furthermore, for **2**, a forbidden half-field transition was observed, which is characteristic of Δ*M*_*S*_ = ± 2 for antiferromagnetic exchange interaction of dimers of Cu­(II). These affirmations align with the ESR spectra area ratio and line width as the temperature decreases. The IR, absorption spectra, and magnetic studies of **1**–**3** were analyzed and compared to those of analogous complexes with the same geometry.

## Graphical abstract

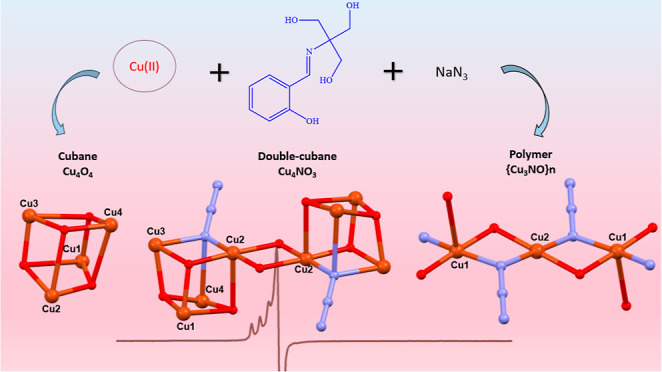

## Introduction

The synthesis and characterization of polynuclear complexes with paramagnetic metal ions have been the subject of intense studies since the discovery of their remarkable biological, catalytic, and magnetic properties.^1–3^ Particularly, Cu­(II) complexes are interesting from both structural and functional perspectives. The stereochemical diversity of Cu­(II) compounds resulted in the formation of numerous mononuclear and polynuclear complexes with multiple topologies, starting from polydentate Schiff bases used as building blocks.^4–6^

The focus of current research in the field of magnetochemistry,^7^ bioinorganic modeling,^8^ catalysis,^9^ and multielectron transfer studies is on tetranuclear Cu­(II) complexes.^10^ There is a wide variety of structures among Cu_4_: cyclic,^11^ pinwheel,^12^ square planar,^13^ dimeric,^14^ face-to-face,^15^ roof-shaped,^16^ cubane type,^17^ and so on.^10^ Hydroxo-, alkoxo-, and phenoxo-bridged Cu­(II) complexes involving a {Cu_*n*_O_*n*_} core (*n* = 2, 4, 6, etc.) are extensive in the literature, primarily due to their significance in molecular magnetism and the comprehension of exchange interactions between metal centers bridged by heteroatoms.^9,18^ These studies offer insights into the structural characteristics that govern magnetic interactions in polynuclear Cu­(II) complexes, thereby providing a framework for the design of new materials with magnetic properties.^9^ Despite significant advances in magneto-structural analysis, the limits of strict orthogonality remain unresolved and continue to evolve, thereby expanding the potential for ferromagnetic materials at higher exchange angles, and therefore remain an open area of research.^9,19^ On the other hand, due to their structural and magnetic diversities, azido-bridged complexes have also received attention. The versatility and efficiency of the azido anion as a ligand lie in its functionality as a terminal mono-, di-, tri-, or tetradentate bridging ligand.^20^

Many alkoxo-bridged cubane-like complexes based on transition metals have been prepared to date using multidentate ligands with ‘NNO’ or ‘NOO’ donor sets that coordinate to the metal ion.^10^ In these systems, the cubane framework is typically assembled through μ_3_-alkoxido/oxo oxygen bridges, leading to the formation of the {Cu_4_O_4_} core.^21,22^ A search of the Cambridge Structural Database (CSD Version 6.01)^23^ covering the period from 1965 to the present indicates that only a limited number of {Cu_4_O_4_} cubane-type complexes featuring μ_3_-alkoxido bridges have been reported (284 hits).^24–26^ In contrast, mixed μ_3_–O/μ_3_–N cubane-like cores have been described for other metal ions; for example, Christou and co-workers reported complexes containing a {Mn_4_O_3_N} core.^27–29^ However, to the best of our knowledge, no examples of a {Cu_4_O_3_N} cubane-like core have been reported in the CSD to date. This highlights the novelty of the present Cu­(II) system, in which a μ_3_-N vertex is incorporated into an otherwise oxygen-rich cubane framework. We tentatively attribute the rarity of such Cu-based mixed μ_3_–O/μ_3_–N cubanes to the strong preference of Cu­(II) for O-donor bridging environments and to the typical coordination behavior of azide, which more commonly acts as a μ_1,1_ or μ_1,3_ *end-on/end-to-end* linker rather than stabilizing a μ_3_-bridging in Cu­(II) clusters. In this context, the complexes we report explore a quite novel bridging mode of the alkoxo group, and we also report the first structure with a cubane-like core {Cu_4_O_3_N}, including a N site at one of its vertices with Cu­(II) ions.

This work represents an extension of the earlier research on alkoxo-bridged polynuclear transition metal complexes,^30^ and we report herein the synthesis and characterization of [Cu_4_(H_2_L)_4_(H_2_O)] **1**, [Cu_4_(μ_3_-N_3_)­(H_2_L)_2_(HL)­(H_2_O)]_2_ **2**, and [Cu_3_(H_2_L)_2_(μ-N_3_)_2_]_*n*_ **3**, using a potentially multidentate Schiff base ligand (H_4_L), having both bridging and chelating capacities. In a classical {Cu_4_O_4_} cubane, the coordination of the four Cu­(II) centers necessitates the presence of four Schiff bases. In the {Cu_4_O_3_N} core, the N atom functions as an additional connecter donor into the cubane framework, supplying a Schiff base, stabilizing the cluster, and modifying the electronic and magnetic responses.

## Experimental Section

### Materials, Equipment, and Measurement Conditions

Electronic spectra were measured with a Beckman DU Series 7000 spectrophotometer on ca. 10^–4^ M methanolic solutions, at 298 K, in the 200–800 nm range. Infrared spectra (KBr pellets) were measured on a Nicolet Magna-IR 750 spectrophotometer. For polycrystalline powder samples, X-band ESR spectra were obtained with a Bruker ELEXSYS E500 II spectrometer, between 300 and 80 K. The *g-* and *A-*tensor components were obtained by fitting ∼9.4641 GHz spectra recorded at both temperatures. Magnetic measurements were performed in gelatin capsules using a Physical Property Measurement System (PPMS) from Quantum Design, and all data were corrected for diamagnetic contributions from the H_4_L ligand and core diamagnetism calculated using Pascaĺ’s constants.^31^ Measurements were performed in weak magnetic fields, from 20 to 200 G, and in the range of 2.9–300 K, after zero-field cooling (ZFC). Single-crystal X-ray diffraction intensities were collected at 295 K on a Stoe-Stadivari diffractometer, equipped with an Axo microfocus source (Ag Kα radiation, λ = 0.56083 Å) and a Dectris Pilatus-100K detector. The structures were refined with SHELXL and deposited with the CCDC (deposition numbers: CCDC-2463002-2463004). Powder diffraction patterns were recorded on a Stoe-Stadivari diffractometer (Ag Kα radiation, λ = 0.56083 Å, 65 kV, 0.60 mA). The sample was packed in a 0.3 mm Lindemann capillary tube, which was mounted on a goniometer head. X-ray exposition over 6 h was carried out with the tube placed vertically (χ = 0, 2θ = 0.2°) and rotated continuously over φ at 15 deg/s. The sample-to-detector distance was fixed to 110 mm. The resulting frames were processed with the *Poly* module in X-Area^32^ to give 1D patterns in the 2θ range 1–20°. The scattering background was eliminated using a 7-point spline function. Simulated X-ray patterns were calculated with Vesta^33^ for **1** and **3** and Mercury^34^ for **2**, using single-crystal refinements.

The elemental composition was obtained by XPS, using a Surface Science Instruments SSX-100, operated at 2 × 10^–9^ Torr, with monochromatic Al Kα radiation (1486.6 eV), and a 1 mm diameter beam size was used. Photoelectrons were collected at a 55° emission angle. A hemispherical analyzer determined electron kinetic energy, using a pass energy of 150 V for wide/survey scans and 50 V for high-resolution scans. A flood gun was used for charge neutralization of nonconductive samples. The atomic percentages obtained from XPS were calculated from the peak areas using the relative sensitivity factors. Since only one spectrum was acquired, uncertainties were estimated by assuming a relative error of 5%–10% in the peak areas, as is commonly accepted in quantitative XPS analysis.^35,36^

#### Synthesis of 2-[(2-Hydroxybenzylidene)-amino]-2-hydroxymethylpropane-1,3-diol (H_4_L)

The ligand H_4_L was synthesized using our previously reported method.^37^

#### Synthesis of [Cu_4_(H_2_L)_4_(H_2_O)] (1)

This compound has been reported in a previous study;^38^ however, it is important to note that the methodology used by Lazarou et al. in those studies differs from that employed in the present research. A mixture of H_4_L (0.0224 g, 0.1 mmol) and NEt_3_ (56 μL, 0.4 mmol) was dissolved in a methanol/water solution (10 mL), followed by the addition of Cu­(NO_3_)_2_·3H_2_O (0.0484 g, 0.2 mmol). The resulting solution was stirred for 1 h. Subsequently, the solution was filtered and allowed to evaporate slowly at room temperature (Scheme 1). After 2 days, blue crystals of **1**·CH_3_OH were collected by filtration and selected for X-ray diffraction. Yield: 81%, mp 218 °C. FT-IR (KBr, cm^–1^): 3352 (br, m), 1629 (s), 1543 (m), 1303 (m), 1055 (m), 466 (w). λ_max_ (nm) [ε, M^–1^·cm^–1^] (in MeOH), 358 (2488), 630 (45.8). FAB-MS (positive ion mode): *m*/*z* = 286 [Cu­(H_2_L)]^+^ (calc. 286.01); *m*/*z* = 922 [Cu_4_(H_2_L)_3_]^+^ (calc. 922.97); *m*/*z* = 1146 [(Cu_4_(H_2_L)_4_]^+^ (calc. 1146.05). Atomic % from XPS calculated for C_45_H_58_Cu_4_N_4_O_18_: C 45.15%, H 4.88%, N 4.68%, O 24.06%, Cu 21.23%; found: C 44.90%, H 4.58%, N 4.48%, O 24.17%, Cu 21.09%.

**Scheme 1:**
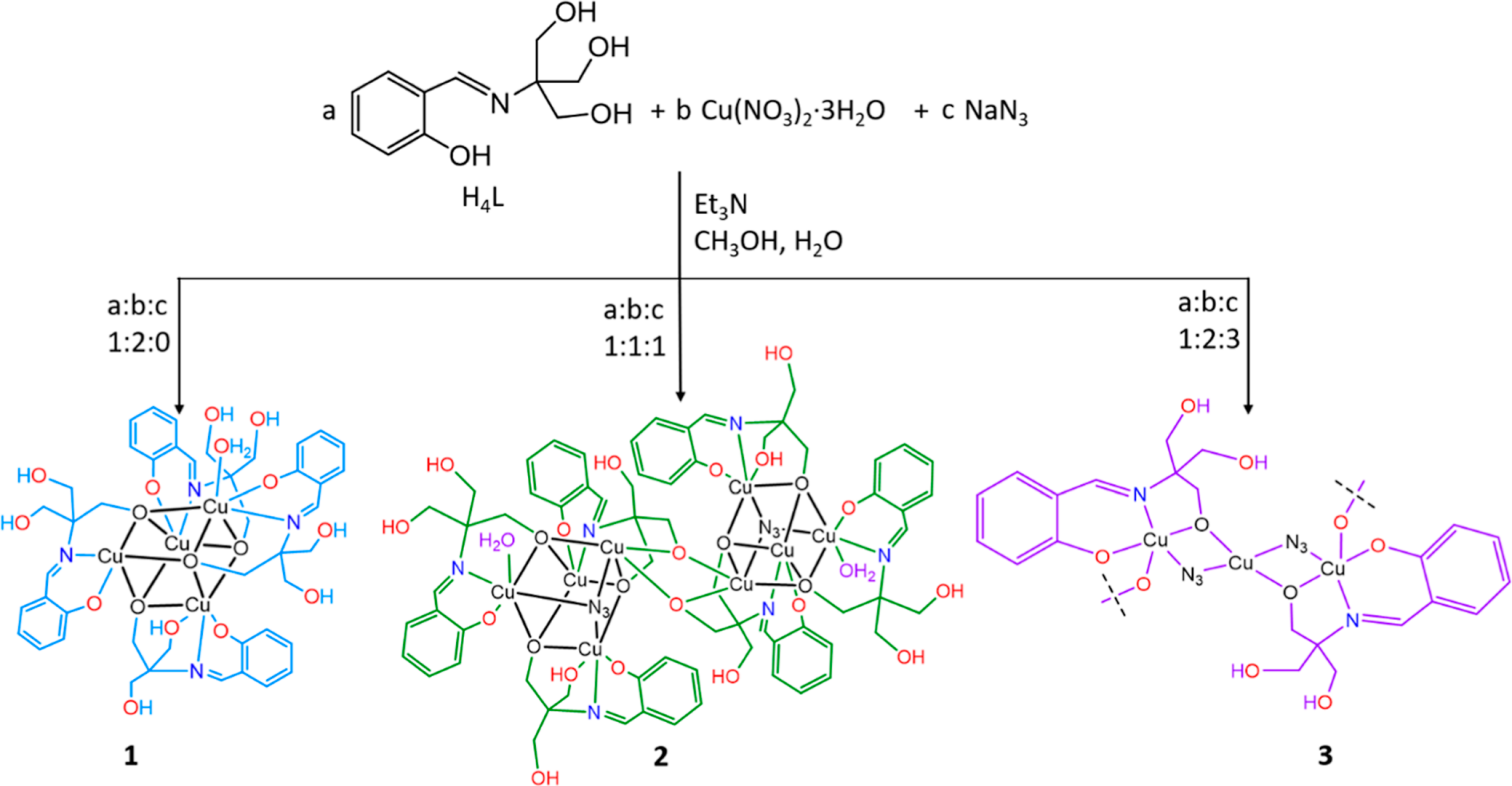
Synthetic Scheme for Complexes **1**–**3** ^a^

a The colors used for bonds are consistent with the colors of crystals.

#### Synthesis of [Cu_8_(H_2_L)_4_(HL)_2_(H_2_O)_2_(κ^**1**^-μ_3_-N_3_)_2_] (2)

A mixture of H_4_L (0.0224 g, 0.1 mmol) and NEt_3_ (56 μL, 0.4 mmol) was dissolved in a methanol/water solution (10 mL), followed by the addition of Cu­(NO_3_)_2_·3H_2_O (0.0242 g, 0.1 mmol) and NaN_3_ (0.0065 g, 0.1 mmol). The resulting solution was stirred for 1 h (Scheme 1). Then, the solution was filtered and slowly evaporated at room temperature, yielding very small crystals. These crystals were recrystallized from ethanol by slow evaporation at room temperature. After 5 days, the formation of green crystals of **2**·C_2_H_5_OH·9.4H_2_O was observed, which were suitable for single-crystal X-ray diffraction analysis. Yield: 53%, mp 142 °C. FT-IR (KBr, cm^–1^): 3388 (br, m), 2083 (m), 1624 (s), 1541 (m), 1384 (m), 1049 (m), 466 (w). λ_max_ (nm) [ε, M^–1^·cm^–1^] (in MeOH), 362 (3350), 615 and 685 (62 and 72). ESI-MS (positive ion mode): *m*/*z* = 366 [Cu_2_(H_2_L)­H_2_O]^+^ (calc. 366.95); *m*/*z* = 1627 [Cu_7_(HL)_2_(H_2_L)_3_N_5_]^+^ (calc. 1628.98); *m*/*z* = 678 [(Cu_3_(H_2_L)_2_N_3_)]^+^ (calc. 679.11). Atomic % from XPS calculated for C_68_H_104_Cu_8_N_12_O_36_: C 37.57%, H 4.82%, N 7.73%, O 26.49%, Cu 23.38%; found: C 37.49%, H 4.64%, N 7.93%, O 26.05%, Cu 24.6%.

#### Synthesis of *catena*-poly­[Cu_3_(H_2_L)_2_(κ^**1**^-μ_2_-N_3_)_2_]_n_ (3)

A mixture of H_4_L (0.224 g, 1 mmol) and NEt_3_ (112 μL, 0.8 mmol) was dissolved in a methanol/water solution (10 mL), followed by the addition of Cu­(NO_3_)_2_·3H_2_O (0.484 g, 2 mmol) and NaN_3_ (0.195 g, 3 mmol). The resulting solution was stirred for 1 h, and then it was filtered and slowly evaporated at room temperature (Scheme 1). After 5 days, green crystals of **3** were collected by filtration. Yield: 53%, mp 200 °C. λ_max_ (nm/ε, [M^–1^·cm^–1^]) (in DMSO): 446/1277, 615/294, and 692/305; IR (KBr, cm^–1^): 3184 (ν_O–H_), 1622 (ν_C=N_), 1290 (ν_C–O_), 3437 and 2092 (ν_N3_^–^), 472 (Cu–O). FAB-MS (positive ion mode): *m*/*z* = 329 [(Cu­(H_2_L)­(N_3_)]^+^ (calc. 328.02); *m*/*z* = 391 [Cu_2_(H_2_L)­N_3_]^+^ (calc. 390.95); *m*/*z* = 638 [Cu_3_(H_2_L)_2_]^+^ (calc. 922.95). Atomic % from XPS calculated for C_22_H_26_Cu_3_N_8_O_8_: C 36.64%, H 3.63%, N 15.54%, O 17.75%, Cu 26.44%; found: C 37.16%, H 3.28%, N 15.34%, O 17.39%, Cu 26.08%.

## Results and Discussion

### Synthesis (Scheme 1)

The reaction between H_4_L and Cu­(NO_3_)_2_·3H_2_O and Et_3_N as base, with a stoichiometric ratio H_4_L:Cu = 1:2, affords compound **1**, which was crystallized as a methanol monosolvate. In this compound, for each Cu­(II) ion, there is one polydentate (H_2_L)^2–^ ligand, which is coordinated to the metal ion. Initially, a H_4_L:Cu:NaN_3_ = 1:2:2 stoichiometric ratio was probed, with the hope of introducing (N_3_)^−^ as a potentially bridging ligand. However, this ligand did not coordinate, and only **1** was recovered.

By changing the stoichiometric ratio while maintaining the same reaction conditions, we obtained other complexes with different nuclearities (**2** and **3**). The novel compound **2** was obtained as green crystals with a ratio of H_4_L:Cu:NaN_3_ of 1:1:1. Lattice solvent molecules were present in the crystals after recrystallization from ethanol. The structure of the compound under consideration contains eight Cu­(II) ions, which are bridged by oxygen atoms and κ^1^ *-μ*_3_-N_3_-azide groups coordinated in the *end-on* mode. For this reaction, adding an excess of Et_3_N resulted in more hydroxy groups of H_4_L being deprotonated, giving a formula that includes both (H_2_L)^2–^ and (HL)^3–^ ligands, as well as (N_3_)^−^. However, the charge balance is consistent with Cu­(II) ions as metal centers. For **3**, the stoichiometric ratio H_4_L:Cu:NaN_3_ was finally modified to 1:2:3, respectively, in a methanol solution, and this change led to the formation of a polymeric compound, with trimeric Cu­(II) units bridged by *end-on* azido ligands. In this case, (H_2_L)^2–^ ligands are found in the complex, and the excess of NaN_3_ used in this reaction favors polymerization.

The formation of different nuclearities under closely related conditions indicates that the assembly process is susceptible to a minor alteration in the coordination of the azido ligand, Schiff-base deprotonation, and solvent effects. These factors influence the connectivity between Cu­(II) centers, resulting in either discrete cubane units or extended architectures. They also provide useful guidelines for the design of related systems with other metal ions.

### Description of Structures

The molecular and crystal structures of **2**·C_2_H_5_OH·9·4H_2_O and **3** obtained from single-crystal X-ray diffraction data (Table S1) are shown in Figures 1 and 3. Details of the molecular structure of **1** have been reported previously, but this report provides an improved refinement.^38^ Experimental PXRD patterns for compounds **1**–**3** have been added to the Supporting Information (Figures S1–S3), and they agree with the corresponding simulated patterns, confirming phase purity of the bulk samples used for magnetic and ESR measurements.

**Figure 1:**
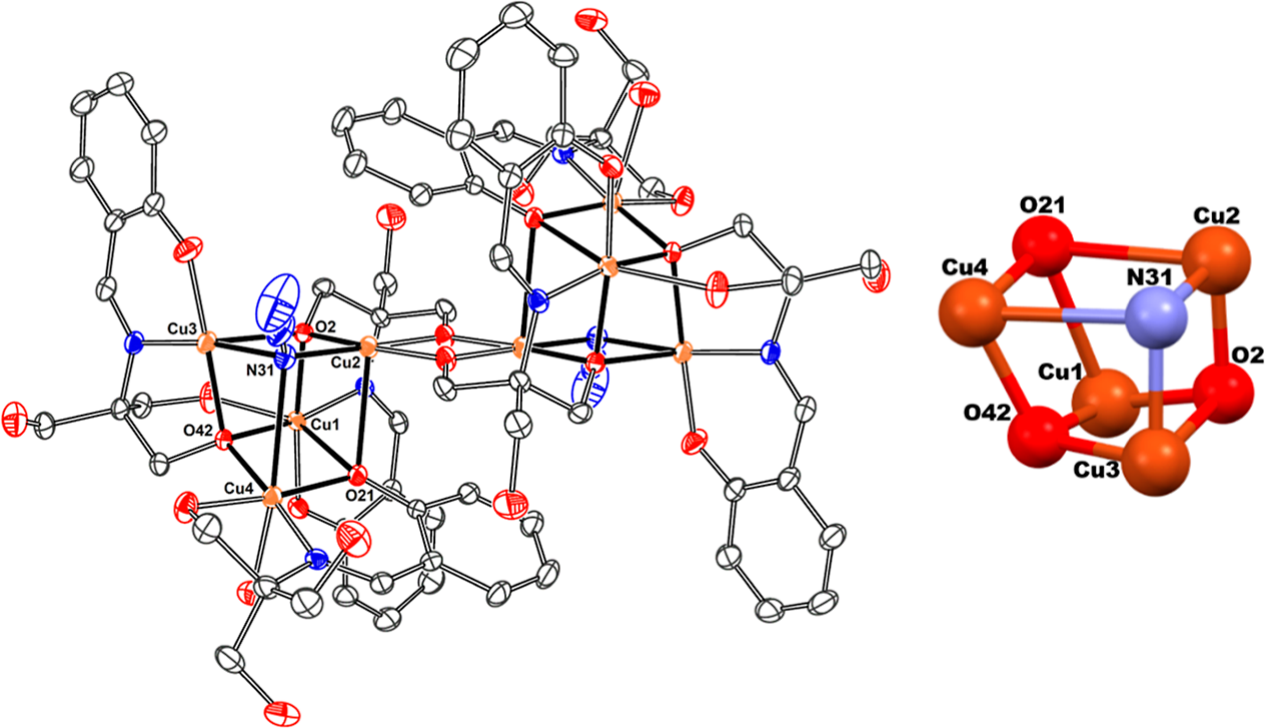
Left panel: Molecular structure of **2**, with ellipsoids at the 10% probability level. Hydrogen atoms are omitted for clarity. Right panel: Geometry of one crystallographically independent cubane cluster.

**Figure 2:**
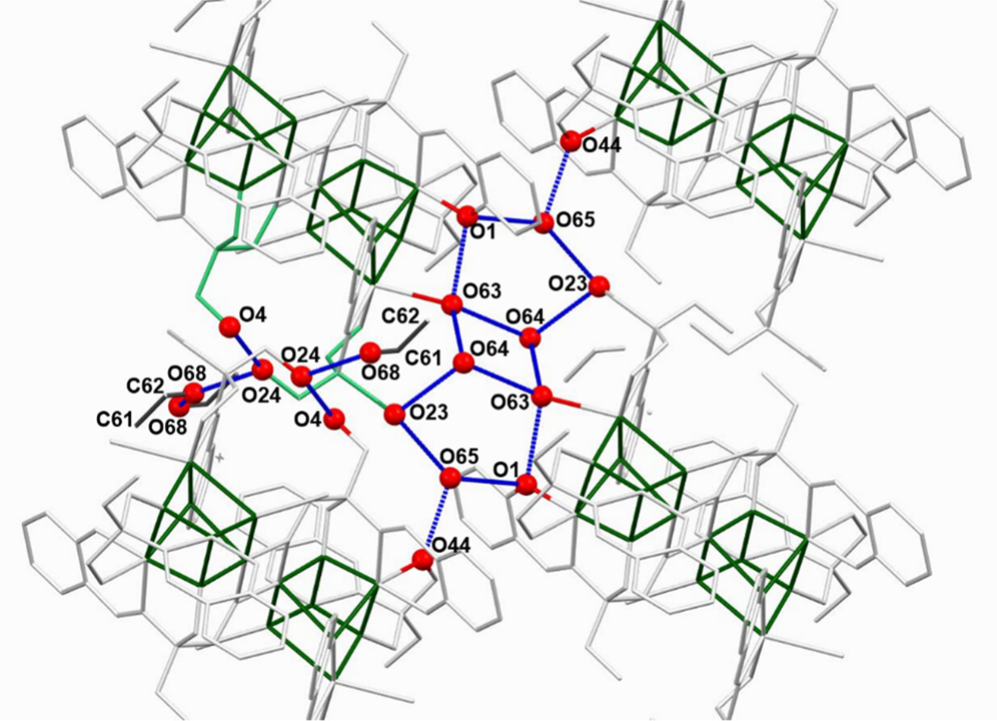
Supramolecular arrangement of hydrogen-bonded molecules in **2**, emphasizing hydrogen bonds originating from water and ethanol solvent.

Compound **2**, which crystallizes in space group *P*1̅, has a double-cubane structure, where {Cu_4_O_3_N} cubes are connected through a centrosymmetric double bridge, using deprotonated hydroxyl groups of (HL)^3–^ ligands (Figure 1). Within each cubane, the four Cu centers exhibit different coordination spheres and geometries. Nonmetallic vertices in the cubane are μ_3_-O atoms from alkoxo or phenoxo groups and one κ^1^ *-*μ_3_-N_3_ from the azido ligand. The resulting coordination geometry for Cu1/Cu4 is octahedral, while Cu2/Cu3 has a square pyramidal geometry. Same features as for **1** are observed: a strong Jahn–Teller for octahedral Cu­(II) sites,^39^ although less pronounced, and a slight trigonal distortion of penta-coordinate sites, with τ_5_ = 0.109 and 0.113 (Figure S4).^40^

The Cu···Cu separations are in the range of 3.0391(6) to 3.4559(5) Å within a cubane, and a shorter Cu···Cu distance is observed for the intercubane bridge, 2.9584(6) Å. Angles for bridges Cu–O–Cu in the cubane range from 74.43(7) to 108.9(1)°, while Cu–N_azido_–Cu angles span the smallest range, from 86.3(1) to 101.7(1) ° (Table S2).^41^ The presence of ethanol and water in the lattice of **2** allows the formation of hydrogen-bonded clusters of solvent molecules, which connect dicubane complexes. For example, a framework based on pentagonal and square rings sharing edges is formed along direction [100] (tFigure [Fig fig2], Table S3).^42,43^

Complex **3** crystallizes in a monoclinic cell with space group *P*2_1_/*n*. The asymmetric unit contains half of a trinuclear cluster, with the central atom, Cu2, placed on an inversion center. Neighboring Cu­(II) atoms are double-bridged by an azido ligand in the *end-on* coordination mode and one deprotonated alkoxo group of the (H_2_L)^2–^ Schiff-base ligand. This monomeric unit polymerizes, through the formation of a long (2.701 Å) intermolecular O···Cu bond, involving one protonated alkoxo group in (H_2_L)^2–^. Since polymerization occurs on both sides of the monomer, a diperiodic polymeric framework is formed (Figure 3), parallel to the (001) crystallographic plane. Atom Cu1, involved in polymerization, exhibits a square pyramidal pentacoordinate geometry: the tridentate (H_2_L)^2–^ and the bridging azido ligands are positioned in the basal plane, while the hydroxy group occupies the apical position, generating the polymeric framework. The central atom in the monomer, Cu2, has a square planar, centrosymmetric coordination geometry. Therefore, it presents a perfect square plane geometry.^44^

**Figure 3:**
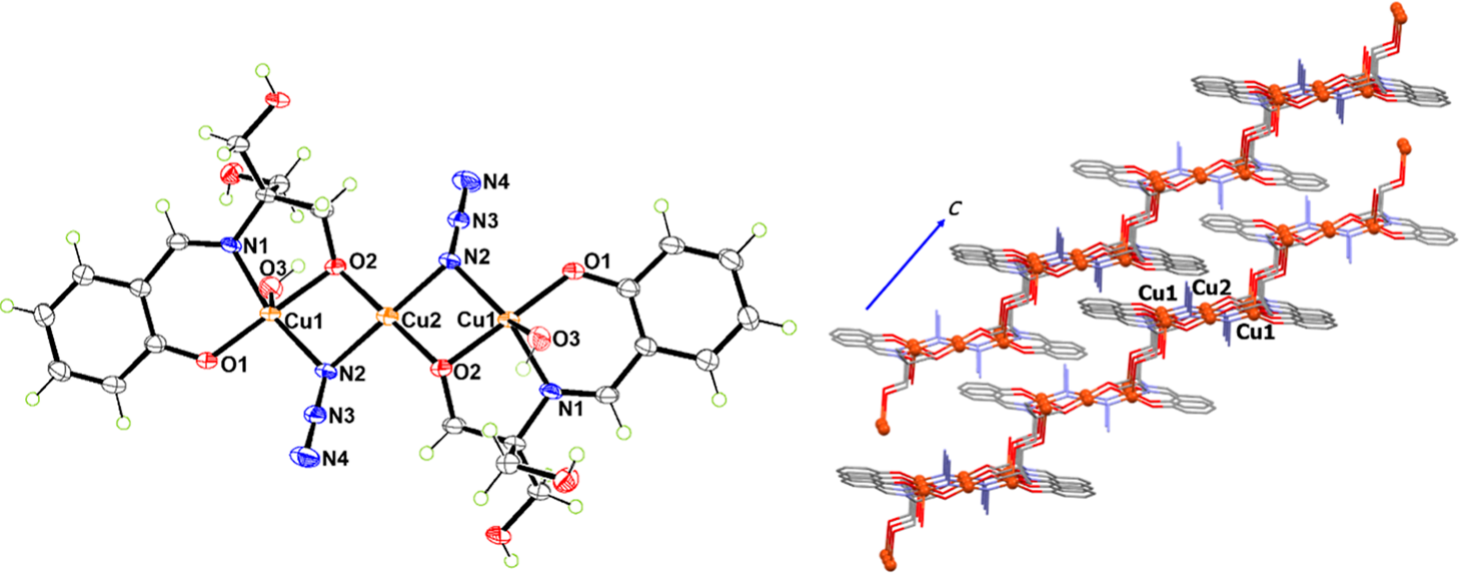
(Left) Monomeric structure of **3**. ORTEP diagram with displacement ellipsoids at the 50% probability level. (Right) The 1D polymerization of the complex along the *b*-axis.

The angles around bridging alkoxo and azido O/N atoms are Cu1–O2–Cu2 = 98.87(10)° and Cu1–N2–Cu2 = 94.84(11)°; they are a little larger than expected for compounds with alkoxo and azido bridges (Table S4).^45^ The Cu···Cu separation within the monomeric unit is the shortest observed for the herein reported complexes, 2.929 Å. Any other Cu···Cu distance in the 2D polymer is much larger (>6 Å), because of the steric volume of (H_2_L)^2–^ ligands.^46^

### Infrared and Electronic Spectral Studies

The absorption spectra of **1**–**3**, measured in methanol at room temperature, show the expected electronic transitions for the Schiff base ligand (H_2_L)^2–^ and (HL)^3–^ (Figure 4).^37^ The hypsochromic shifts of the allowed transitions π → π* and *n* → π* due to coordination to Cu­(II) are observed. LMCT transitions are assigned to the bands with less intensity at 358 (**1**), 362 (**2**), and 425, 446 nm (**3**).^47^ Bands with λ_max_ between 615 and 692 nm are assigned to the forbidden *d–d* transitions for symmetry, which have small ε values and are broad, corresponding to highly distorted octahedral geometries, in accordance with X-ray structures.^48^ In **1**, at 630 nm, the transition is assigned to ^2^ *E*_*g*_ *→* ^2^ *T*_2*g*_. For **2** and **3** at 615 nm, the band is assigned to the *d*_*xz*_, *d*_*yz*_ *→ d*_*x*_^2^_*–y*_^2^ (^2^ *B*_1*g*_ →^2^ *E*_*g*_) transition for both compounds. Bands at 685 and 692 nm are assigned to *d*_*z*_^2^ *→ d*_*x*_^2^_*–y*_^2^ (^2^ *A*_1*g*_ →^2^ *E*_*g*_) transitions, respectively (Diagrams S1 and S2).^49–51^

**Figure 4:**
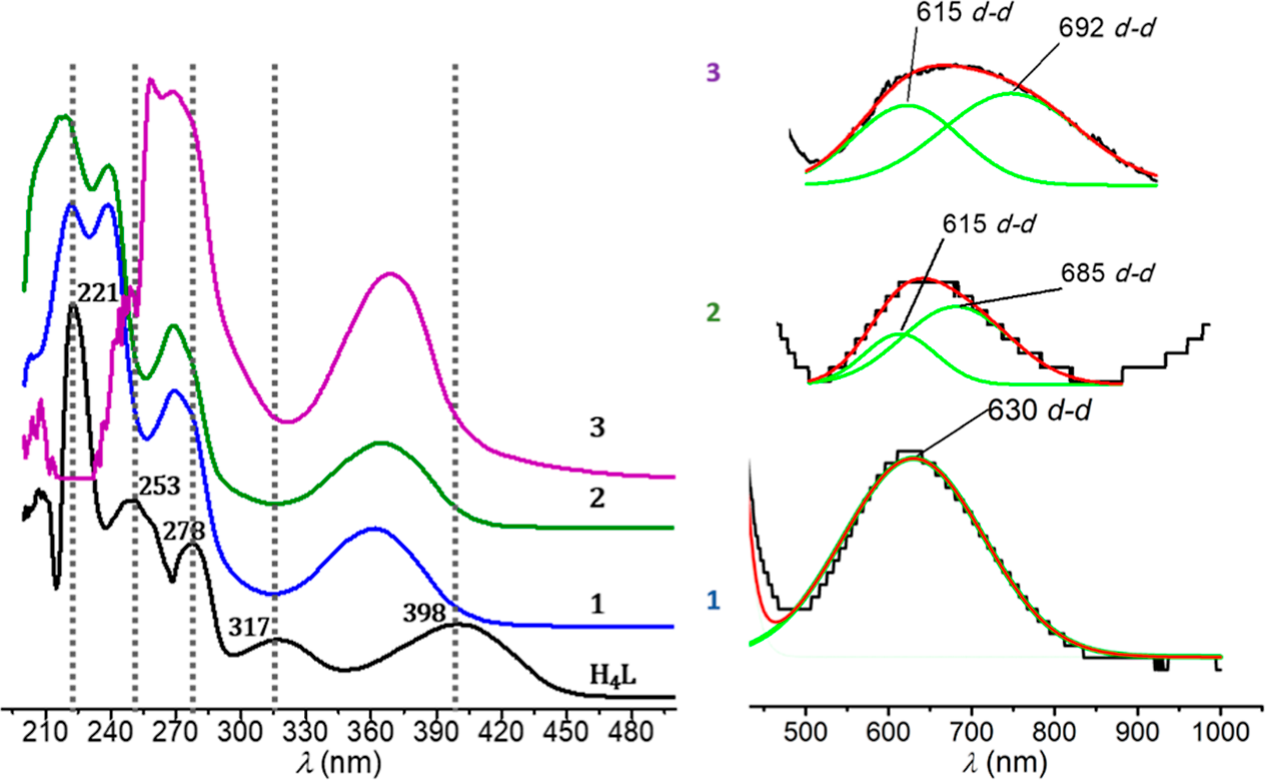
Left: Electronic spectra and λ­(*nm*)/ε­(*M* ^–1^·*cm* ^–1^) values for the H_4_L ligand: 221/2082, 253/1058, 278/837, 317/310, 398/394; **1**: 224/10831, 241/10741, 273/5965, 358/2545, **2**: 216/16512, 240/14470, 269/8168, 362/3447, and **3**: 258/43799, 270/42419, 302/11608, 368/22527, respectively. Right: *d–d* transitions for **1**–**3**. The green lines are used for deconvolving *d–d* transitions.

### IR Spectra

In the IR spectra of **1**–**3** (Figure S5), some shifts in the absorption frequencies are observed for important functional groups in (H_2_L)^2–^ and (HL)^3–^, compared to H_4_L, because of coordination. The OH aliphatic vibrations are shifted to higher energy, and the C–O stretching vibration of the phenolic group appears at lower frequencies, compared to the free ligand.^30,37^ The coordination of the N atom of the imine bond is also confirmed, since the C=N stretching band is observed at lower frequencies, which corresponds to a reduction of the double bond character for this group (Table S5). The bands observed at 466 and 472 cm^–1^ are attributed to Cu–O and Cu–N bonds, respectively.^52^ Finally, a broad band in the range of 3352–3184 cm^–1^ confirms the presence of water in **1** and **2**, whether it coordinates to Cu­(II) or stabilizes the crystal structure in the form of a hydrate. All IR data suggest that the azido bridge is present only in compounds **2** and **3**.^30^

### ESR Studies

The ESR spectra of polycrystalline samples of **1**–**3** were measured at 90 and 300 K. The calculated *g*-values provide information about oxidation and spin states of the metal ions, as well as whether the magnetic structure around the metal ions changes.^53^ In **1** at 300 K, an axial spectrum was observed, with *g*_⊥_ = 2.0305, *g*_∥_ = 2.2999, and *S* = 1/2, and a zero-field splitting was observed at 460 G. Upon cooling to 90 K, the zero-field signal and the *g* values remained unchanged. At the same time, the hyperfine splitting became more clearly resolved, arising from coupling Ŝ·Î (Figure 5).^53^ The hyperfine coupling constant is *A*_Cu_ = 197 × 10^–4^ cm^–1^, which agrees with hyperfine constant values for Cu­(II) compounds with distorted octahedral geometry.^54,55^ The area ratio $\frac{A_{300K}}{A_{90K}}=1.49<3.33$ suggests the presence of antiferromagnetic exchange interactions.^56^ However, the change in line width, given by the values of Γ_300*K*_ = 272 and Γ_90*K*_ = 248 G, decreased at lower temperatures. The values of the area ratio and line width suggest the presence of both antiferromagnetic and ferromagnetic exchange interactions in these conditions.^57^

**Figure 5:**
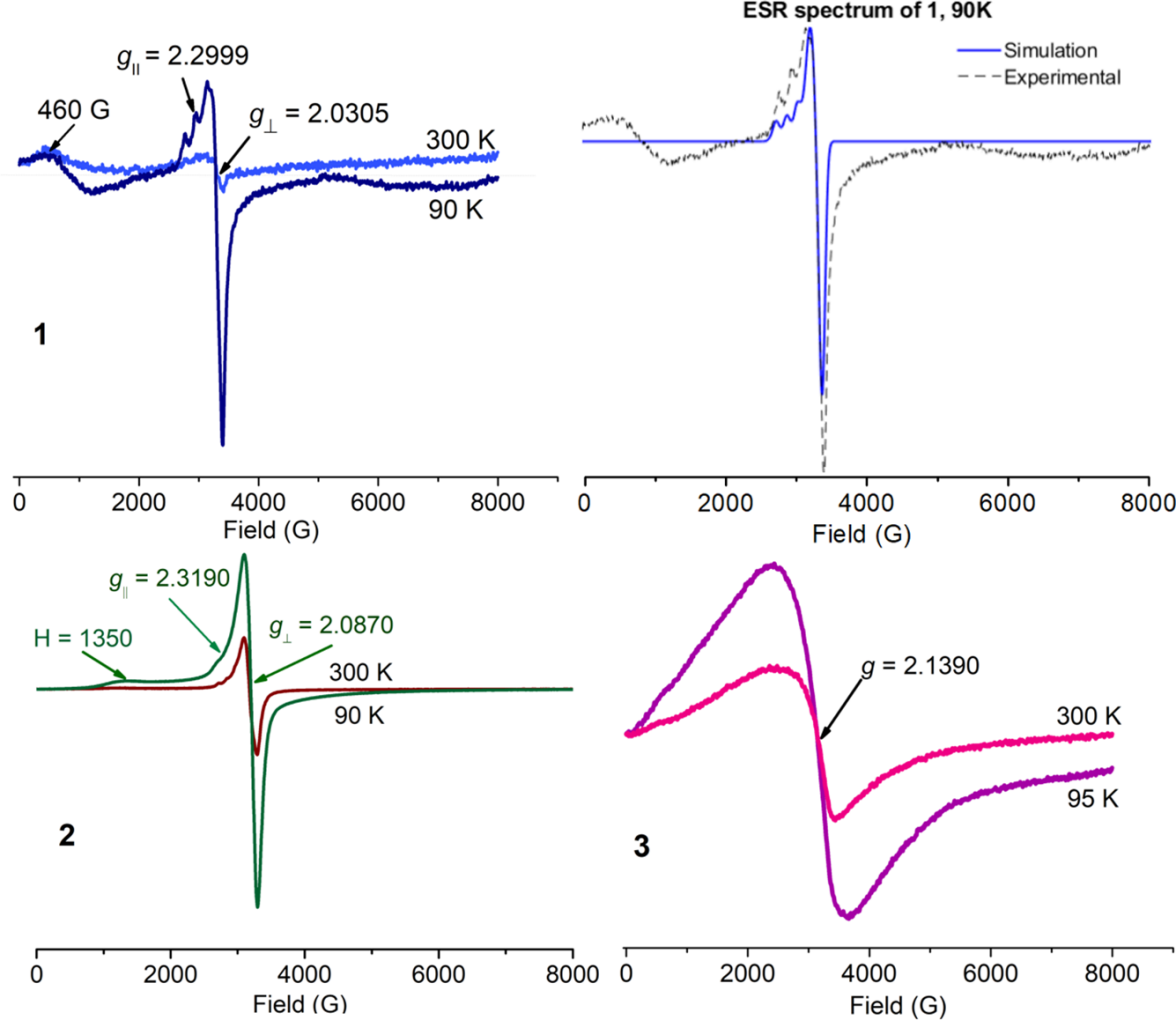
Experimental ESR spectra for complexes **1**–**3** measured at 300 and 90 K and simulated using EasySpin for **1**; for **2**, and **3** are in Supporting Information (Figures S6–S8).

The full Hamiltonian corresponding to this system is

1 $$Ĥ=D\left[{Ŝ}_{z}^{2}‐\frac{1}{3}S\left(S+1\right)\right]+g\beta_{e}\mathrm{H⃗}\cdot {Ŝ}_{z}-g_{N}\beta_{N}\mathrm{H⃗}\cdot {Î}_{z}+A{Î}_{z}\cdot {Ŝ}_{z}-JS_{1}\cdot S_{2}$$

where the first term is zero-field splitting, the second and third terms are the Zeeman nuclear and electronic splitting, the fourth term is the *hf*, and the last one is the magnetic exchange interaction.^48^

The ESR study of **2** (Figure 5) revealed an axial spectrum with *g*_∥_ = 2.3190 and *g*_⊥_ = 2.0870, indicative of *S* = 1/2.^56^ Furthermore, the half-field signal at ∼1350 G is well-resolved and corresponds to the forbidden transition with Δ*M*_*s*_ = ± 2 for antiferromagnetic exchange in Cu­(II) dimers.^52,58^ The area ratio, $\frac{A_{300K}}{A_{90K}}=14.16>3.33$ , and the decrease in line width, from *Γ*_300 *K*_ = 192 G to *Γ*_90 *K*_ = 112 G, suggest ferromagnetic exchange interaction.^57^ Measurements carried out in DMSO at 90 K afforded an axial spectrum with hyperfine splitting, *g*_∥_ = 2.2555, *g*_⊥_ = 2.0521. The constant for hyperfine coupling is *A*_Cu_ = 172 × 10^–4^ cm^–1^, typical of Cu­(II) compounds with distorted octahedral geometry (Figure 5).^59^ The spin Hamiltonian used for the interpretation is the same as for **1** (eq 1). The main difference between **1** and **2** is thus that the zero-field splitting observed in the former is not present in the latter.

The ESR spectra of **3** exhibited a broad singlet (Figure 5), characteristic of compounds with dipolar magnetic interactions, which are common in polymeric Cu­(II) compounds.^60^ The *g* = 2.1390 value for *S* = 1/2 is expected for Cu­(II) compounds. Since the *g* value remains constant from 300 to 95 K, this indicates that the magnetic structure is maintained over this temperature range. As in **2**, the area ratio $\frac{A_{300K}}{A_{95K}}=4.24>3.15$ ,^55^ and the narrowing of the line width (Γ_300*K*_ = 2292 G and Γ_90*K*_ = 1400 G), when the temperature is lowered, suggests the presence of ferromagnetic exchange interactions, although dipolar interactions dominate.^61^ When measured in DMSO at 77 K, an axial spectrum with *hf* interaction signals is observed, with values of *g*_∥_ = 2.2692 and *g*_⊥_ = 2.0487 (Figure S9).^62^ The ESR spectra in DMSO imply magnetic and molecular dilution by diminishing the concentration of these. These effects allow the spectra of some samples to provide more information, including hyperfine structures. In solution, the species are more discrete and molecular and differ completely from the crystallographic arrangement in the solid state.

This could be a consequence of the weakness of Cu1–O3 bonds (2.701(3) Å, Table S4).^63^ Furthermore, the *hf* coupling is also observed with four signals and *A*_Cu_ = 169 × 10^–4^ cm^–1^. The *g* values of **3**, both in the solid state and in solution, are in accordance with literature data for Cu­(II) compounds with *D*_4h_ symmetry.^61,64^ Additionally, these values of *g*_∥_>*g*_⊥_ > 2 suggest that, in the ground state, the unpaired electron occupies the $d_{x^{2}-y^{2}}$ orbital.^62,65^ The spin Hamiltonian describes the ESR spectrum of **3** in the following form:

2 $$Ĥ=\mathrm{D̂}\left[{Ŝ}_{z}^{2}‐\frac{1}{3}S\left(S+1\right)\right]-g\beta g_{N}\beta_{N}\left[\frac{Ŝ\cdot Î}{r^{3}}‐\frac{3\left(Ŝ\cdot \mathrm{r⃗}\right)\left(Î\cdot \mathrm{r⃗}\right)}{r^{5}}\right]+A{Î}_{z}\cdot {Ŝ}_{z}-JS_{1}\cdot S_{2}$$

Compared with the previous Hamiltonian, the added terms are the dipole interactions and the exchange interactions that may be present due to spectrum broadening.^62,66^

The ESR spectra in DMSO were simulated using the EasySpin 6.0.8 package,^67^ and the best fits between the experimental and simulated results are listed in Table 1 (Figures S6–S10). For axial spectra, the parameter *G* quantifies the exchange interaction between the copper centers and is defined as *G* = (*g*_∥_–2.0023)/(*g*_⊥_+2.0023).^68,69^ Values of *G* > 4 suggest negligible exchange interaction, whereas values below 4 indicate significant exchange interaction within the complex.^68^ The parameter α^2^ represents a measure of covalency, describing the *in-plane* sigma bonding that originates from the dipole–dipole interactions between magnetic moments. These magnetic moments are associated with the electron spin motion and the nucleus. It is important to note that this value decreases with increasing covalency.^58,69^ The ESR parameters *g*_∥_, *g*_⊥_, and *A*_∥_(Cu), as well as the energies of *d–d* transitions, were utilized to determine the bonding parameters α^2^, β^2^, and γ^2^, which may be considered as indicators of the covalency in *in-plane* σ-bonds, *in-plane* π-bonds, and *out-of-plane* π-bonds.^69,70^ Furthermore, the orbital reduction factors *K*_∥_ and *K*_⊥_ were also determined.^69^ Consequently, the geometric parameter *G* and the α^2^ values are determined from all simulated spectra. The calculation of these parameters is achieved through the implementation of the subsequent eqs 3–7.^68,69^

3 $$\alpha^{2}=\left(\frac{A_{∥}}{0.036}\right)+\left(g_{∥}-2.0023\right)+\frac{3}{7}\left(g_{⊥}-2.0023\right)+0.04$$

4 $$K_{∥}^{2}=\left(g_{∥}-2.0023\right)E_{d-d}/8\lambda_{0}$$

5 $$K_{⊥}^{2}=\left(g_{⊥}-2.0023\right)E_{d-d}/2\lambda_{0}$$

6 $$K_{∥}=\alpha^{2}\beta^{2}$$

7 $$K_{⊥}=\alpha^{2}\gamma^{2}$$

In accordance with Hathaway’s findings,^71^ when the pure σ-bonding is formed, the *K*_∥_≈*K*_⊥_≈0.77, but when *in-plane* π-bonding, *K*_∥_<*K*_⊥_, whereas for *out-of-plane* π-bonding, *K*_∥_>*K*_⊥_.^69,72^ For **1**–**3**, it is the first case, while the bonding parameters, α^2^, β^2^, and γ^2^ < 1, affirm the covalent character of the chelate ligand. The spin–orbit coupling constant is assigned to a value of −828 cm^–1^ for the Cu­(II) *d* ^9^ system.^72^ Therefore, the values of α^2^ indicate that approximately 80% of the total spin population is found in the $d_{x^{2}-y^{2}}$ orbital of **1**–**3**.^60,69^ In Table 1, the spin Hamiltonian and bonding parameters of **1–3** are summarized.

**Table 1:** Spin Hamiltonian and Bonding Parameters of Complexes **1**–**3**

solid state at 300 K									
***g***_∥_ **and** *g*_⊥_	2.2999, 2.0305 (Complex **1**)								
	2.3190, 2.0870 (Complex **2**)								
***g***_**iso**_	2.1390 (Complex **3**)								
**DMSO solution at 90 K**									
	***g***_∥_	***g***_⊥_	***G***	***E***_***d‑d***_ **cm** ^**–1**^	***A***_∥_ **cm** ^**–1**^ **(10** ^**–4**^ **)**	**α** ^2^	***K***_∥_	***K***_⊥_	***K*** ^2^
**1** simulated for two species	2.2923	2.0505	6.01	15,874	153	0.776	0.597	0.496	0.695
	2.2715	2.0102	5.58	15,874	157	0.749	0.576	0.479	0.645
**2**	2.2555	2.0521	5.08	14,599	172	0.792	0.610	0.507	0.558
**3**	2.2692	2.0487	5.75	14,451	170	0.799	0.615	0.511	0.582

### Magneto-Structural Correlations

The magnetic susceptibility at variable temperature for **1**–**3** was performed on air-dried powdered samples in the 2.9–300 K range with an applied field of 1000 Oe. Curves of χ_M_ *T* are shown in Figure 6. At 300 K, χ_M_ *T* for **1** is 3.2 cm^3^·mol^–1^·K, higher than the value of 1.5 cm^3^·mol^–1^·K expected for four Cu­(II) ions (*S*_*T*_ = 2) that do not interact magnetically with each other. Upon cooling, the value of χ_M_ *T* decreases almost linearly to reach a plateau of ∼2.6 cm^3^·mol^–1^ K at 35–85 K. Finally, the product χ_M_ *T* decreases to 2.2 cm^3^·mol^–1^·K at 2 K, showing that antiferromagnetic exchange interactions increase as *T* decreases; however, there is no change in magnetic structure from ferro to antiferro, with ferromagnetic exchange interactions dominating over antiferromagnetic exchange interactions up to 2 K.^26,73^

**Figure 6:**
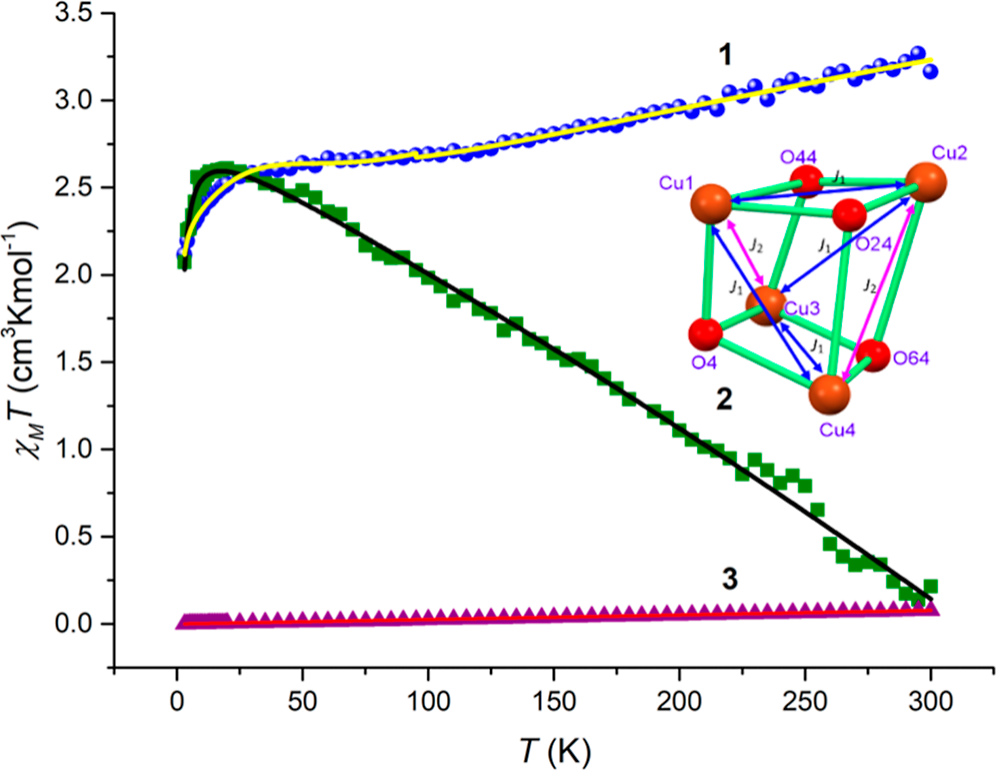
χ_M_T vs T for complexes **1** (blue circles), **2** (green squares), and **3** (purple triangles). Solid lines represent the best fit of the experimental data of this work. Inset: Assuming the interaction occurs through the short Cu–O bonds (lime bonds), the coupling scheme (*J*_1_ and *J*_2_) proposed for **1** is shown.

The magnetic characterization of **2** is shown in Figure 6 (green squares), plotting χ_M_ *T* vs *T*. At 300 K, the χ_M_ *T* = 0.21 cm^3^·K·mol^–1^ is lower than the expected value for eight noninteracting Cu­(II) ions (*S*_*T*_ = 4), 3.00 cm^3^·K·mol^–1^. As the temperature decreased, the χ_M_ *T* value gradually increased, reaching a maximum of 2.61 cm^3^·K·mol^–1^ at 21 K, indicating a weak ferromagnetic behavior which is no greater than antiferromagnetic exchange interactions, and then decreased sharply to 2.07 cm^3^·K·mol^–1^ at 2 K. The overall shape of the χ_M_ *T* vs *T* plot of **2** indicates ferromagnetic and antiferromagnetic exchange interactions between the paramagnetic centers within the cubane-like core.^74^

For complex **3**, χ_M_ *T* decreases gradually on decreasing the temperature, from 0.078 cm^3^·mol^–1^·K at room temperature (value expected for three noninteracting Cu­(II) ions: 1.125 cm^3^·mol^–1^·K, assuming *g* = 2 and *S*_*T*_ = 3/2 (Figure 6)). The orbital contributions of the Cu­(II) centers to the magnetic moment are responsible for *g* values that significantly derive from 2.^48^ The value of χ_M_ *T* decreases continuously on cooling, reaching 7.8 × 10^–4^ cm^3^·mol^–1^·K at 2 K. This behavior suggests a dominant antiferromagnetic exchange interaction. The magnetic susceptibility data were fitted using the Curie–Weiss law, yielding a Curie constant of 8.56 × 10^–8^ cm^3^·mol^–1^·K and a Weiss temperature of −0.623 K. The negative Weiss temperature further confirms the presence of antiferromagnetic exchange interactions within the studied range.^48,75,76^

For ferromagnetic cubane **1**, a sound magneto-structural correlation may be drawn based on the variation of the magnetic susceptibility over the 2 to 300 K temperature range. Hatfield and co-workers have proposed magneto-structural correlations for Cu­(II) complexes with hydroxo- and μ-alkoxo-bridges.^77,78^ A structural analysis employing the CSD reveals the presence of three distinct categories of cubane structures, and they are classified depending on long and short Cu···Cu distances.^10,79^ Compound **1** belongs to the second class ‘4 + 2’ (Figure 7).^78,79^

**Figure 7:**
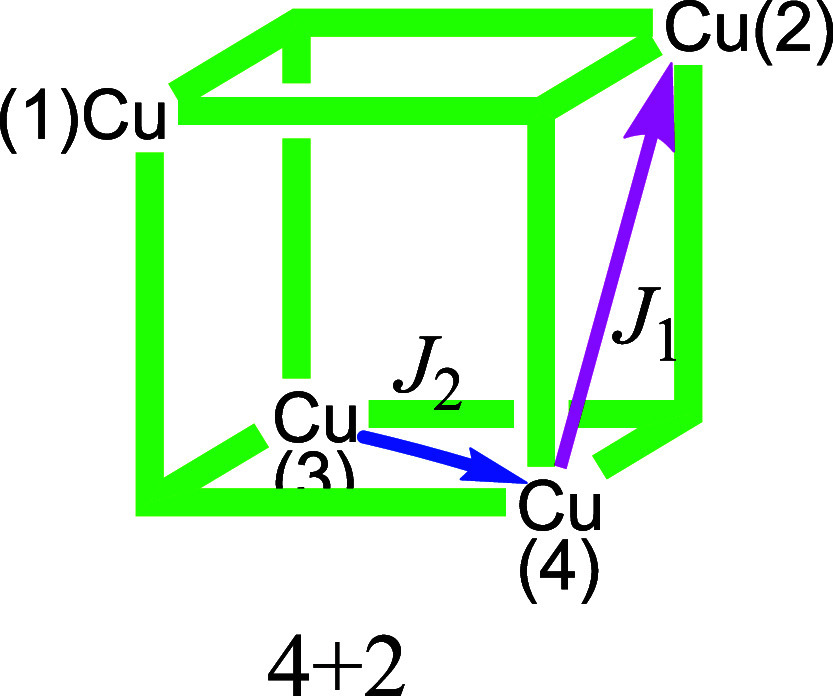
Simple model (*J*_1_, *J*_2_) for the {Cu_4_O_4_} core in complex **1** according to the classification 4 + 2.

Given that metallic centers are placed on the vertices of a tetrahedron, there are six possible exchange interactions between the Cu­(II) ions. In the case of a ‘4 + 2’ cubane, magnetic data can then be fitted with a model including two *J* parameters (Figure 7): for cubane **1**, *J*_1_ is the exchange interaction constant associated with two long distances, Cu2···Cu4 and Cu1···Cu3, while *J*_2_ corresponds to the constant for four short distances, Cu1···Cu4, Cu3···Cu4, Cu2···Cu3, and Cu1···Cu2.^38,78,80^

Therefore, the magnetic data were analyzed using the spin Hamiltonian^78^

8 $$Ĥ=-2J_{1}\left(S_{Cu2}S_{Cu4}+S_{Cu1}S_{Cu3}\right)-2J_{2}\left(S_{Cu1}S_{Cu4}+S_{Cu3}S_{Cu4}+S_{Cu2}S_{Cu3}+S_{Cu1}S_{Cu2}\right)$$

The following expression for molar magnetic susceptibility χ_*M*_ can be derived from the Van Vleck equation, contemplating the following parameters: temperature-independent paramagnetism (TIP), paramagnetic impurities (fraction ρ), and the Weiss constant (θ) (eq 9).^81^

9 $$\chi_{M}=\frac{g^{2}N\beta^{2}}{3k\left(T‐\theta \right)}\frac{A}{B}\left(1‐\rho \right)+\rho \frac{g^{2}N\beta^{2}}{2kT}+TIP$$

where

10 $$\frac{A}{B}=\frac{2\exp\left(\frac{J_{1}}{kT}\right)+\exp\left[\frac{{2J}_{1}‐J_{2}}{kT}\right]+5\exp\left[\frac{{2J}_{1}+J_{2}}{kT}\right]}{1+6\exp\left(\frac{J_{1}}{kT}\right)+\exp\left[\frac{{2J}_{1}‐{2J}_{2}}{kT}\right]+3\exp\left[\frac{{2J}_{1}‐J_{2}}{kT}\right]+5\exp\left[\frac{{2J}_{1}+J_{2}}{kT}\right]}$$

Fit of the experimental data was performed using *g* = 2, with best-fit values of *J*_1_ = −35 cm^–1^ and *J*_2_ = 117 cm^–1^ (Figure 6). The magnetic couplings demonstrate a high degree of congruence with the structural characteristics and the previously reported magneto-structural correlations in {Cu_4_O_4_} systems.^77,82^ For the ‘4 + 2’ class of {Cu_4_O_4_} cores, the Cu–O–Cu angles and Cu–O distances are crucial geometric parameters defining *J* values and the magnetic susceptibility.^80,83^ Alkoxo-bridged complexes demonstrate a comparable tendency: the greater the Cu–O–Cu angle, the more pronounced the antiferromagnetic coupling. Consequently, ideal cubanes with Cu–O–Cu angles approaching 90° are promising candidates for ferromagnetic materials.^10,77,78^

For compound **1**, metrics involving Cu sites with a long separation, which are associated with the setting value of *J*_1_, are Cu2–O24–Cu4 = 100.72(6)°, Cu2–O64–Cu4 = 99.59(6)° and Cu2–O64,24 = 2.641(1), 1.939(1) Å and Cu4–O64,24 = 1.931(1), 2.601(1) Å. For the set of short metal–metal separations, geometric parameters associated with *J*_2_ are Cu1–O24–Cu4 = 87.82(6)°, Cu1–O4–Cu4 = 109.39(8)°, and Cu1–O24,4 = 1.929(2), 1.939(1) Å, Cu4–O4,24 = 1.956(2), 2.601(1) Å.^38^ Tercero et al. have reported that for a compound having an idealized cubane-type structure with Cu­(II) ions bridged by alkoxy groups, the Cu–O distance must be 2.0 Å to give a calculated exchange coupling constant of 16.7 cm^–1^.^84^ When the Cu–O–Cu angle approaches 90°, the magnetic orbitals become almost perpendicular, thereby minimizing the antiferromagnetic superexchange pathway and potentially resulting in dominant ferromagnetic coupling (*J* > 0). As the Cu–O–Cu angle increases (typically >95–100°), there is an increase in orbital overlap, which favors antiferromagnetic superexchange and results in *J* < 0. Therefore, in compound **1**, the Cu–O–Cu angles <90° rationalize the larger ferromagnetic exchange interaction *J*_2_ (117 cm^–1^), while the angles >100° account for the antiferromagnetic exchange interaction *J*_1_ (−35 cm^–1^); therefore, *J*_total_ = *J*_1_ + *J*_2_ = 82 cm^–1^.^75,85^

The measurement of *M* vs *H* at 3K was performed, where the field varied from −4000 to 4000 Oe. A closed hysteresis with a slope of ∼45°, of a magnetically hard material, whose hysteresis does not saturate in this magnetic field window; that is, the magnetic polarization independent of the field was not observed (Figure 8). However, the coercive field of 1 Oe was observed, which is indicative of spins canting. Spin saturation for such systems cannot be observed under the limited measurement conditions available with the used magnetometer. Therefore, the observed magnetization is found to be directly proportional to the externally applied field.^8,86^

**Figure 8:**
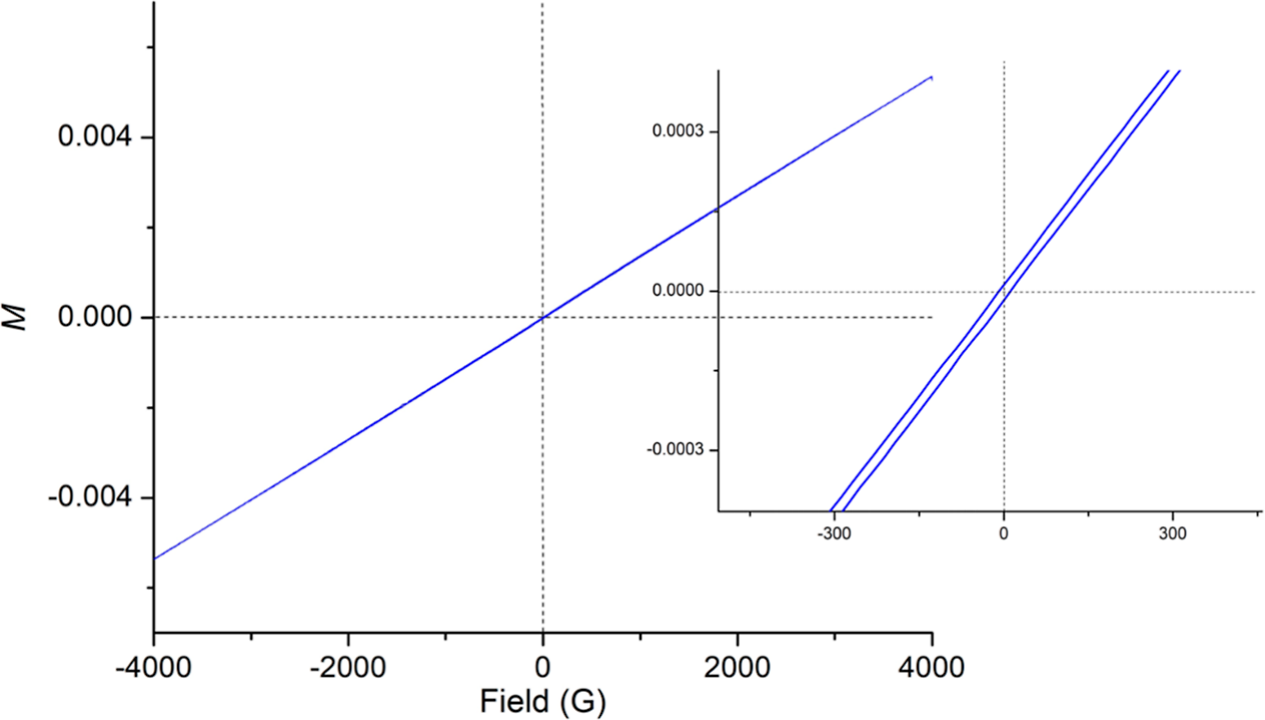
M-H data at 3 K for the {Cu_4_O_4_} core in **1**.

## Conclusion

After an exhaustive search of scientific information, we believe that this paper is the first to report an octa-nuclear Cu­(II) complex containing a {Cu_4_O_3_N} **2** cubane core with μ_3_-alkoxido and μ_3_-azido bridges. Two other compounds, tetra-**1** and tri-**3**, which are tetranuclear and trinuclear, are also reported. The magnetic behavior of **1**–**3** revealed a general antiferromagnetic Cu–O–Cu exchange interaction driven mainly by dominant coupling via alkoxo bridges with angles 90° < Cu–O–Cu ≲180°, as confirmed by the *J* value. As reported, the *end-on* azido bridging, Cu–N­(azido)–Cu, resulted in a ferromagnetic exchange interaction response, consistent with the observed Cu–N­(azido)–Cu ∼ 90° bond angles and the positive *J* value. This ferromagnetism, attributed to the specific geometry of the azido bridges and their contribution to the magnetic exchange interactions, and the intramolecular ferromagnetic exchange interactions within the Cu­(II) pairs, observed in ESR, informed us that the orthogonality of the magnetic orbitals, in at least one pathway of the exchange interaction, led to the observed ferromagnetic and antiferromagnetic coupling. This research highlights the crucial role that sodium azide incorporation, combined with the use of a Schiff base ligand, plays in controlling the formation of tetramers, trimers, and polymers, significantly impacting both the magnetic properties of the compounds and the design of metallic clusters. Overall, the work presented not only deepens the understanding of the correlation between the molecular structure and magnetic behavior but also provides insights into the design of copper-based materials with expected magnetic properties.

## Supplementary Material



## Notes

Crystallography data, PDRX spectra simulated and experimental, including figures of structures, tables of angles, and bond length for the compounds, and additional information about energy values of electronic transitions, FT-IR spectra, and ESR simulated spectra for all compounds in the polycrystalline sample and in DMSO (PDF)
