# Supplementary material for: Tri‑, Tetra‑, Octa-Nuclear Copper Complexes Including the First Mode Cubane-like {Cu4O3N} Core: Synthesis, Structure, and Magnetic Properties
Source: ACS Omega. 2026 Jun 18;11(25):36596–607. doi: 10.1021/acsomega.5c11893 (PMC13325364; doi:10.1021/acsomega.5c11893)
Supplement: Supplementary file 1 [file ao5c11893_si_001.pdf]

Supporting Information

Tri-, tetra-, octa-nuclear copper complexes including the first mode cubane-like $\{Cu_4O_3N\}$ core: synthesis, structure and magnetic properties

Cándida Pastor-Ramírez[1]; Sylvain Bernès[2], Rafael Zamorano-Ulloa[3]; Daniel Ramírez-Rosales[3]; Samuel Hernández-Anzaldo[1]; Yasmi Reyes-Ortega[1*].*

List of content

Table S1. Crystallographic data, details of data collection, and structure refinement parameters for **1**-**3**.
Figure S1. Comparison of the experimental PXRD pattern (blue) with the simulated pattern (black) calculated from single-crystal X-ray diffraction data for **1**.
Figure S2. Experimental(green) and simulated (black) PXRD patterns of **2**. Minor differences in relative intensities may arise from preferred orientation or slight sample crystallinity effects.
Figure S3. Experimental (purple) and simulated (black) PXRD patterns of **3**, showing good agreement between both profiles.
Figure S4. Polyhedral arrangement in **2**, hydrogens omitted for clarity.
Table S2. Selected bond length /Å and angles/° of **2**. Symmetry transformations used to generate equivalent atoms: #1 -x+1, -y+1, -z+1.
Table S3. Hydrogen bond **2** (Å and °).
Table S4. Selected bond lengths (Å) and angles (°) of **3**.
Diagram S1. Energy levels for a Cu(II) ion in crystalline symmetry fields: O_h, D_{4h}, D_{2h}, and C_i.

Diagram S2. MO and energy values of electronic transitions for **1**-**3**.

Figure S5. FT-IR spectra of H_4L and **1**-**3** measured in KBr pellets.
Table S5. Comparison of the infrared bands (cm^{-1}) and the strength constants of the products obtained with the raw material ($x10^5$ dyn/cm).
Figure S6. ESR spectrum simulated and experimental for **1**, in a polycrystalline sample at 90 K.
Figure S7. ESR spectrum simulated and experimental for **2**, in a polycrystalline sample at 90 K.
Figure S8. ESR spectrum simulated and experimental for **3**, in a polycrystalline sample at 95 K.
Figure S9. ESR spectra measured in a DMSO frozen solution of **2** (left) and **3** (right).
Figure S10. ESR spectrum simulated and experimental in DMSO for **2** (left) and **3** (right).

Table S1. *Crystallographic data, details of data collection, and structure refinement parameters for **1-3**.*

Compound	**1** $[Cu_4(H_2L)_4(H_2O)]\cdot CH_3OH$	**2** $[Cu_8(H_2L)_4(HL)_2(H_2O)_2(\mu_3\text{-}N_3)_2]$	**3** $[Cu_3(H_2L)_2(\mu_2\text{-}N_3)_2]_n$
Empirical formula	$C_{45}H_{58}Cu_4N_4O_{18}$	$C_{68}H_{102.8}Cu_8N_{12}O_{35.4}$	$C_{22}H_{26}Cu_3N_8O_8$
Formula weight	1197.11	2163.14	721.13
Space group	$P\bar{1}$	$P\bar{1}$	$P2_1/n$
a, b, c [Å]	10.4216(4), 15.0435(6), 17.7241(7)	12.7227(5), 12.9818([illegible] 14.4796(4)	9.1496(7), 8.6171(8), 16.3740(14)
α, β, γ [°]	67.940(3), 74.336(3), 71.657(3)	91.737(3), 108.275(3), 97.382(3)	90, 94.215(7), 90
V [Å³], Z	2407.79(18), 2	2245.72(13)	1287.48(19)
$D_{calc.}$ [g/cm³]	1.651	1.599	1.860

μ [mm^{-1}]	0.962	1.026	1.328
Reflections	12783 [R_{int} = 0.0443 for 64132 data]	11940 [R(int) = 0.0321for 63583 data]	3476 [R(int) = 0.1139 for 36112 data]
Parameters/restraints	674/12	589/14	198/0
R_1, wR_2 [I > 2σ(I)]	0.0296, 0.0712	0.0431, 0.1202	0.0411, 0.0950
R_1, wR_2 [all data]	0.0538, 0.0829	0.0700, 0.1509	0.0681, 0.1042
Goodness-of-fit on F^2	1.020	1.129	0.905

Table S2. Selected bond length /Å and angles/° of **2**. Symmetry transformations used to generate equivalent atoms: #1 -x+1, -y+1, -z+1.

Cu1–O1	1.917(2)
Cu1–O2	1.955(2)
Cu1–N1	1.955(3)
Cu1–O42#1	2.070(2)
Cu1–O44#1	2.290(3)
Cu1–Cu2#1	3.0391(6)
Cu2–O3	1.906(2)
Cu2–O3#1	1.931(3)
Cu2–O2#1	1.931(2)
Cu2–N31	2.012(3)
Cu2–Cu2#1	2.9584(8)
Cu3–O41	1.894(3)
Cu3–N41	1.920(3)
Cu3–N31	2.017(3)
Cu3–O42	2.040(2)
Cu3–O2#1	2.308(2)
Cu4–O21	1.906(2)
Cu4–N21	1.936(3)
Cu4–O22	1.993(3)
Cu4–O42	2.002(2)
Cu2#1–O2–Cu1	102.91(11)
Cu2#1–O2–Cu3#1	94.51(10)
Cu1–O2–Cu3#1	96.10(9)
Cu2–O3–Cu2#1	100.90(11)
Cu4–O42–Cu3	108.93(11)
Cu4–O42–Cu1#1	104.82(10)
Cu3–O42–Cu1#1	101.35(10)
Cu2–N31–Cu3	101.70(13)

Table S3. *Hydrogen bond **2** (Å and °).*

D–H···A	D(D–H)	D(H···A)	D(D···A)	<(DHA)
O4–H4···O24	0.859(10)	1.99(2)	2.821(5)	164(7)

O23–H23···O64	0.851(10)	2.02(3)	2.806(7)	154(7)
O24–H24···O68#2	0.842(10)	1.95(4)	2.699(10)	148(7)
O44–H44···O65	0.854(10)	1.757(13)	2.610(5)	175(6)
O63–H63A···O64	0.849(10)	1.91(2)	2.732(6)	163(6)
O63–H63B···O1#1	0.849(10)	1.95(3)	2.739(4)	153(6)

Symmetry transformations used to generate equivalent atoms: #1 -x+1,-y+1,-z+1 #2 -x,-y+1,-z+1 #3 -x+1,-y+1,-z #4 -x,-y+2,-z+1

Table S4. *Selected bond lengths (Å) and angles (°) of* ***3***.

Cu1–O1	1.892(2)
Cu1–N1	1.923(3)
Cu1–O2	1.927(2)
Cu1–N2#1	1.997(3)
Cu1–O3#2	2.701(3)
Cu1–Cu2	2.9294(5)
Cu2–O2	1.929(2)
Cu2–O2#1	1.929(2)
Cu2–N2#1	1.982(3)
Cu2–N2	1.982(3)
O1–Cu1–N1	96.62(10)
N1–Cu1–O2	84.59(10)
O1–Cu1–N2#1	96.77(10)
O2–Cu1–N2#1	81.80(10)
O1–Cu1–O3#2	82.35(9)
N1–Cu1–O3#2	103.53(10)
O2–Cu1–O3#2	98.84(9)
N2#1–Cu1–O3#2	86.98(10)
O2–Cu2–N2	97.87(11)
O2#1–Cu2–N2	82.13(11)
Cu1–O2–Cu2	98.87(10)
N3–N2–Cu1#1	124.8(2)
Cu2–N2–Cu1#1	94.84(11)

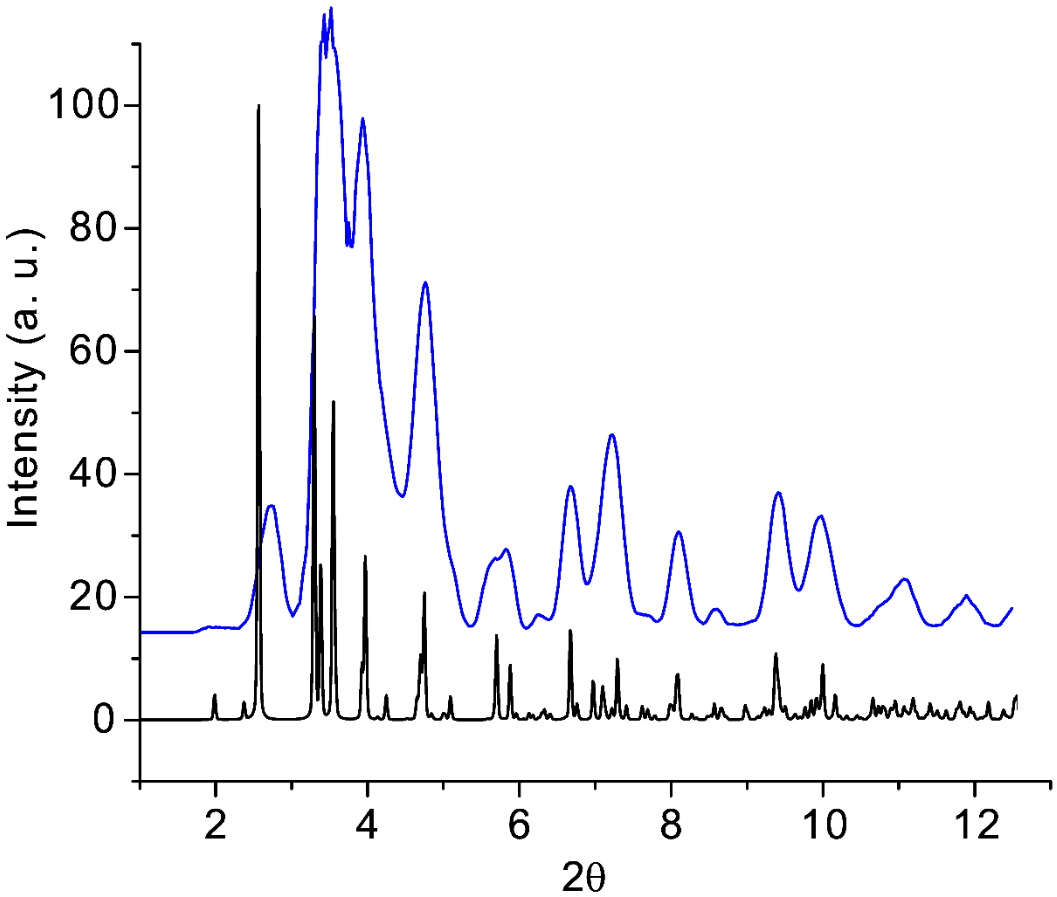

Figure S1*. Comparison of the experimental PXRD pattern (blue) with the simulated pattern (black) calculated from single-crystal X-ray diffraction data for* ***1****.*

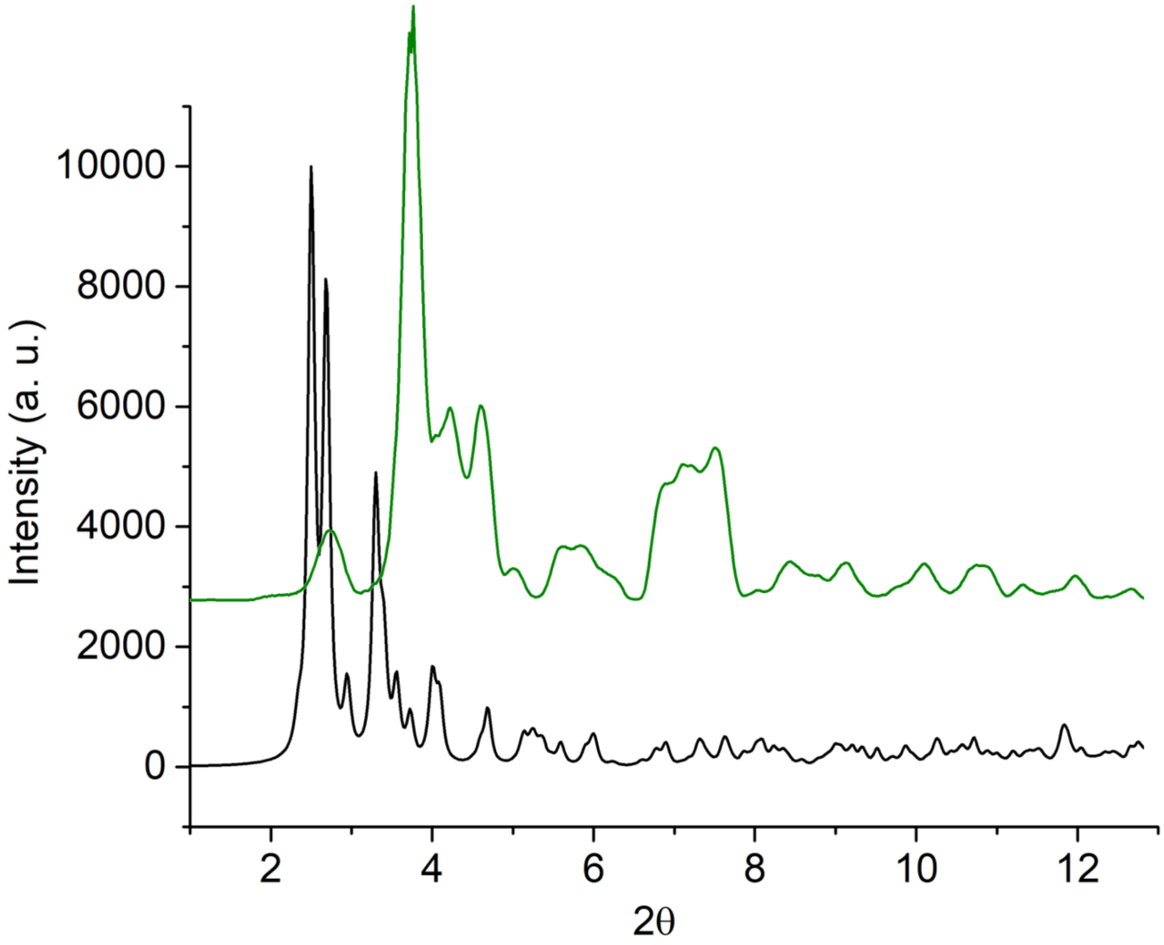

Figure S2. *Experimental(green) and simulated (black) PXRD patterns of* ***2***. *Minor differences in relative intensities may arise from preferred orientation or slight sample crystallinity effects.*

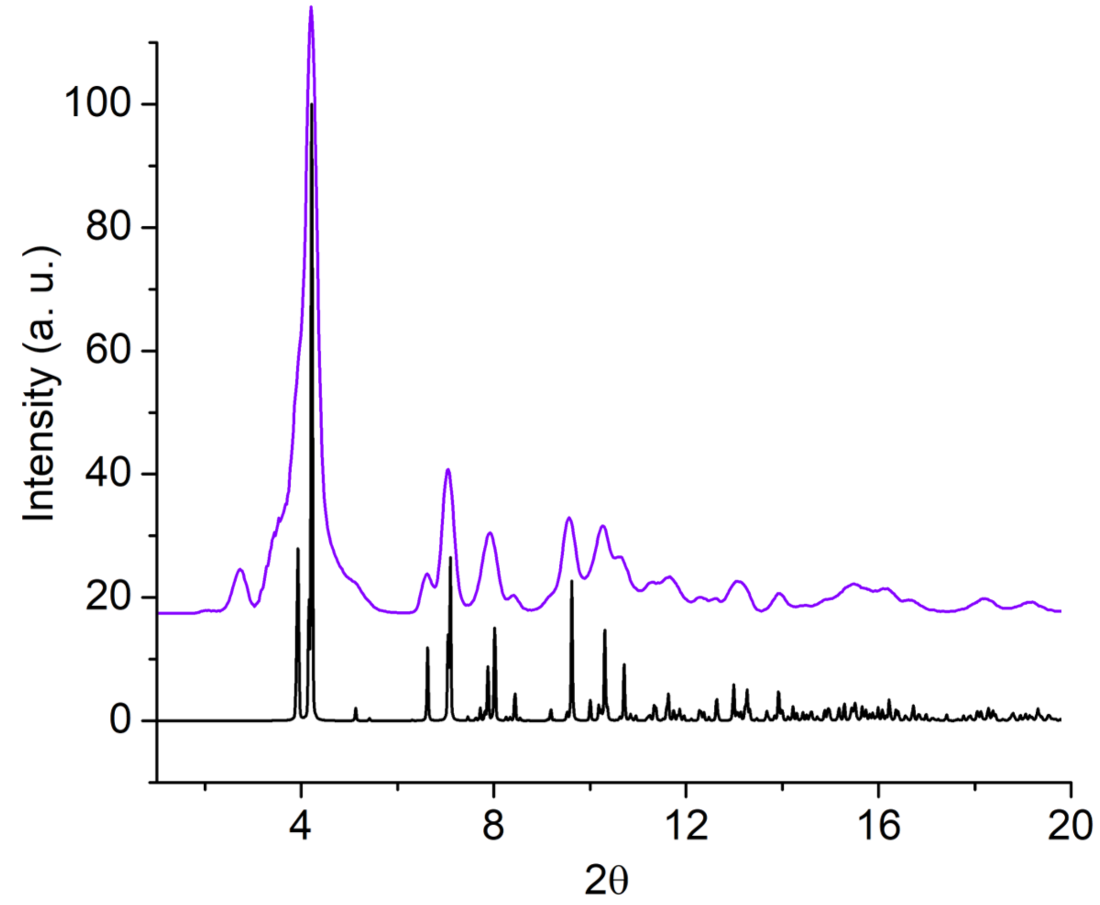

Figure S3. *Experimental (purple) and simulated (black) PXRD patterns of **3**, showing good agreement between both profiles.*

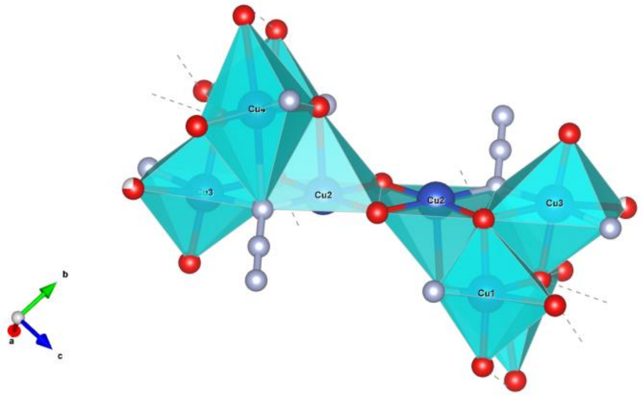

Figure S4. *Polyhedral arrangement in **2**, hydrogens omitted for clarity.*

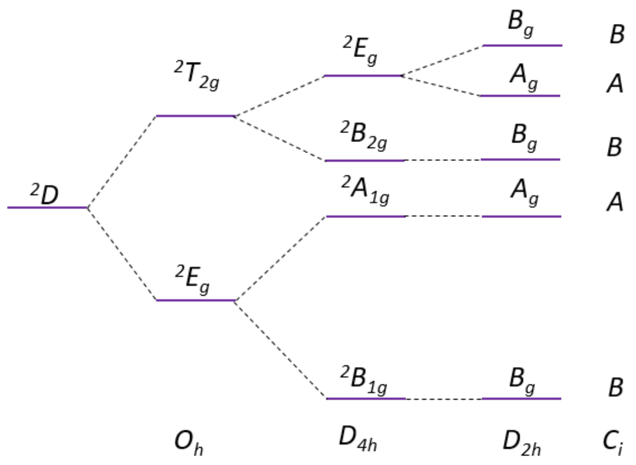

***Diagram S1**. Energy levels for a Cu(II) ion in crystalline symmetry fields: O_h, D_{4h}, D_{2h}, and C_i.*

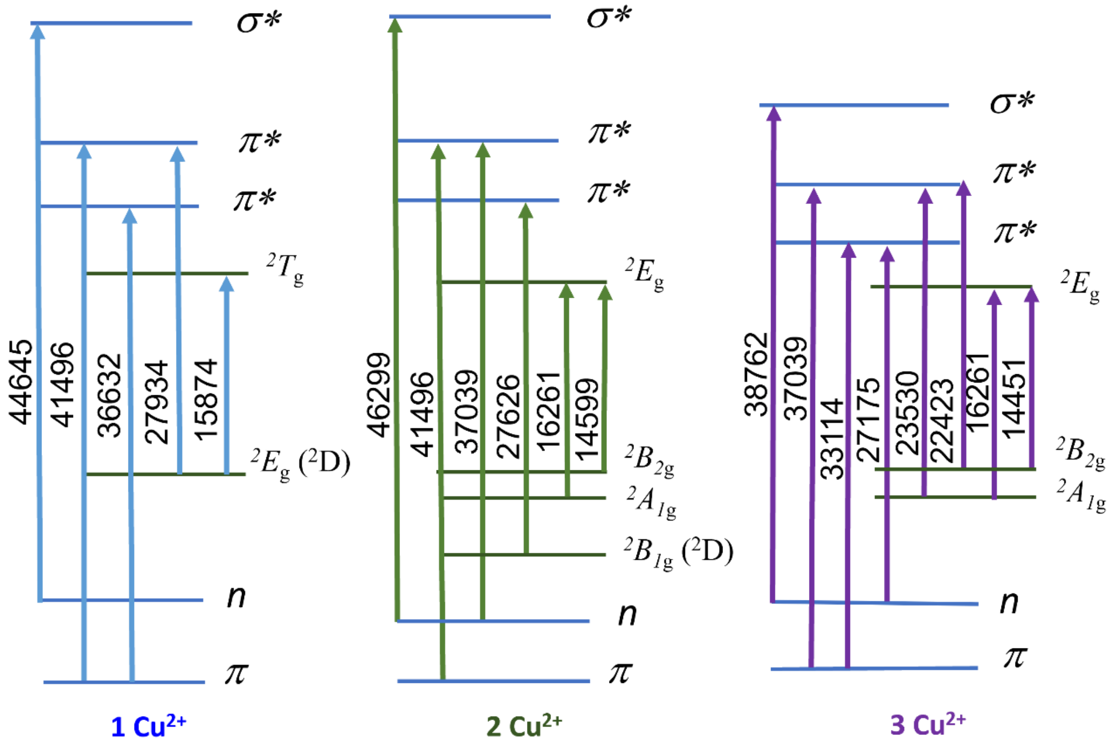

***Diagram S2**. MO and energy values of electronic transitions for **1-3**.*

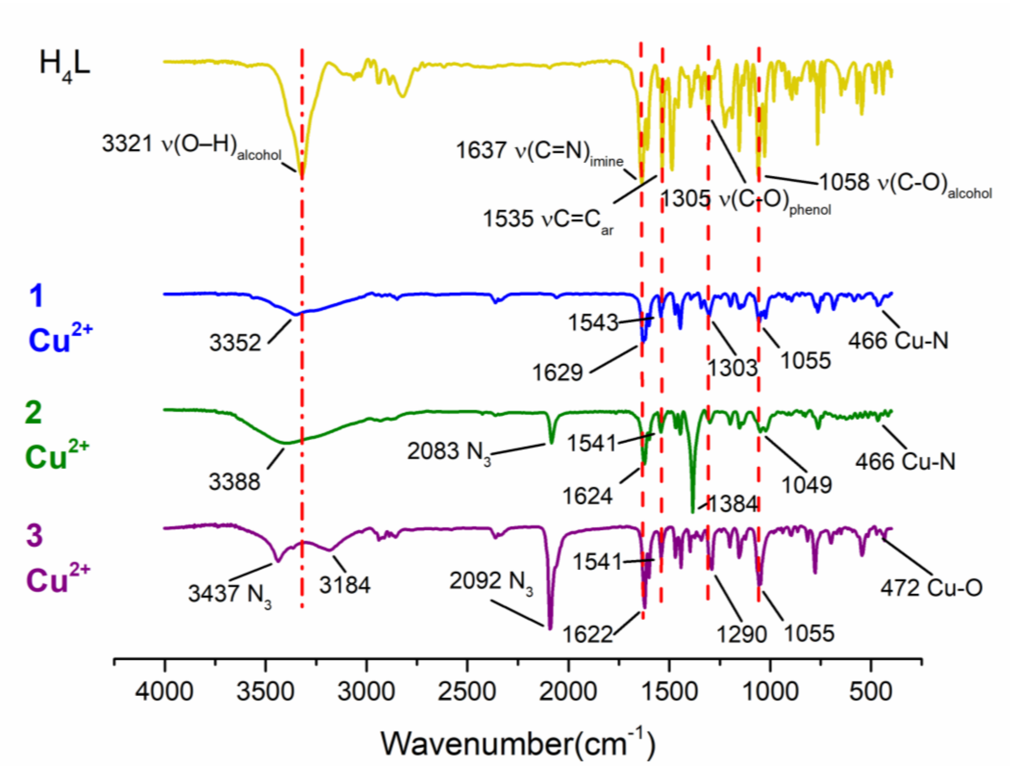

Figure S5. *FT-IR spectra of H_4L and **1-3** measured in KBr pellets.*

Table S5. *Comparison of the infrared bands (cm^{-1}) and the strength constants of the products obtained with the raw material ($x10^5$ dyn/cm).*

Compound	ν_{O-H}/k	$\nu_{(N3^-)asim}/k$	$\nu_{C=N}/k$	$\nu_{C=C}/k$	$\nu_{C-Ofenol}/k$	$\nu_{C-Oalcohol}/k$	M–N y M–O/*k*
H_4L	3321/ 6.15		1637/ 10.19	1535/ 8.33	1301/ 6.83	1058/ 4.52	
**1**	3352/ 6.26		1629/ 10.09	1543/ 8.42	1303/ 6.85	1055/ 4.49	466/ 1.46
**2**	3388/ 6.4	2083/ 17.83	1624/ 10.03	1541/ 8.39	1298/ 6.8	1049/ 4.44	466/ 1.46
**3**	3184/ 5.65	2092/ 18.04	1622/ 10.01	1541/ 8.39	1290/ 6.72	1047/ 4.42	472/ 1.67

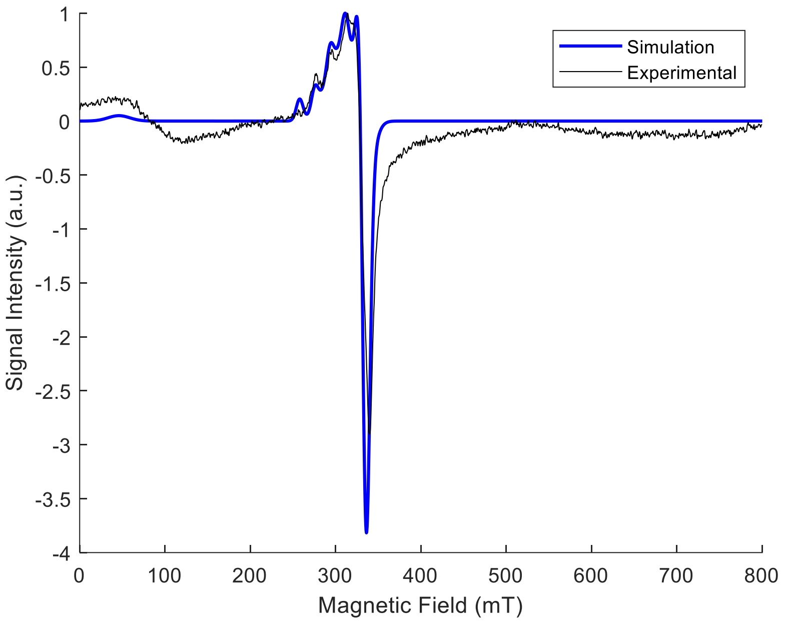

***Figure S6**. ESR spectrum simulated and experimental for **1**, in a polycrystalline sample at 90 K.*

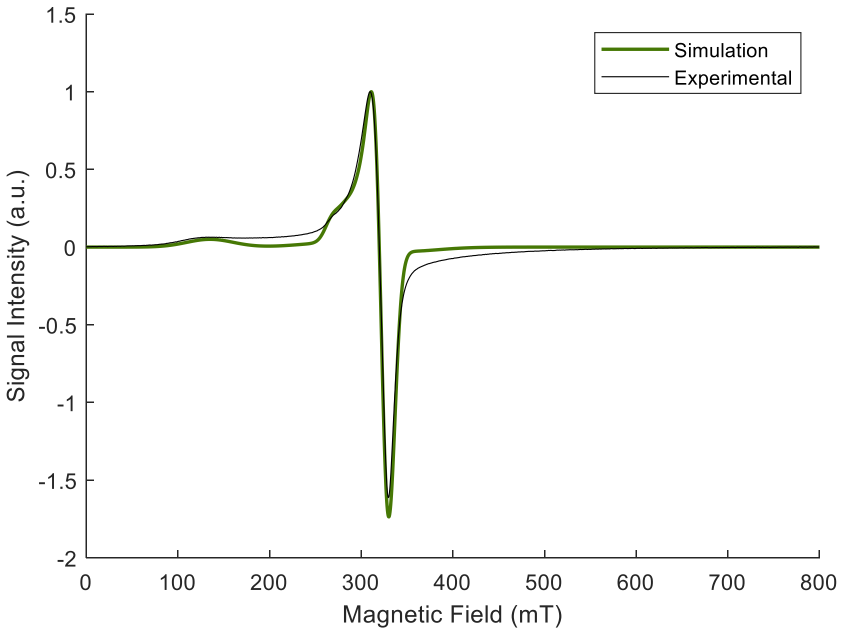

***Figure S7**. ESR spectrum simulated and experimental for **2**, in a polycrystalline sample at 90 K.*

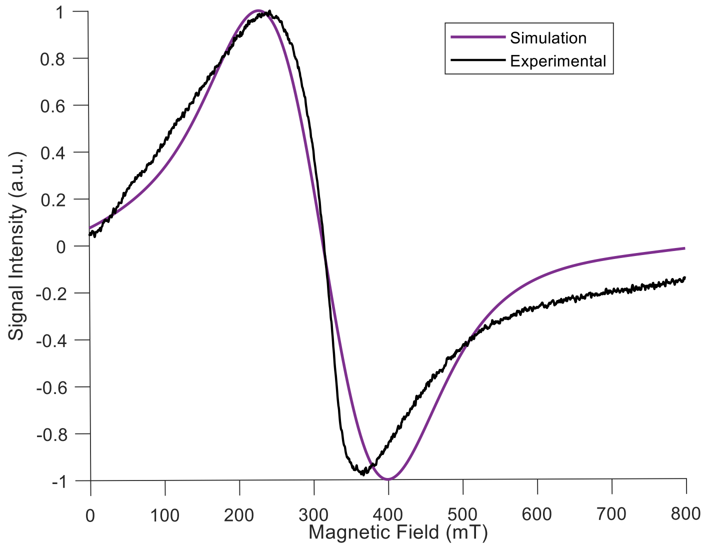

Figure S8*. ESR spectrum simulated and experimental for* ***3****, in a polycrystalline sample at 95 K.*

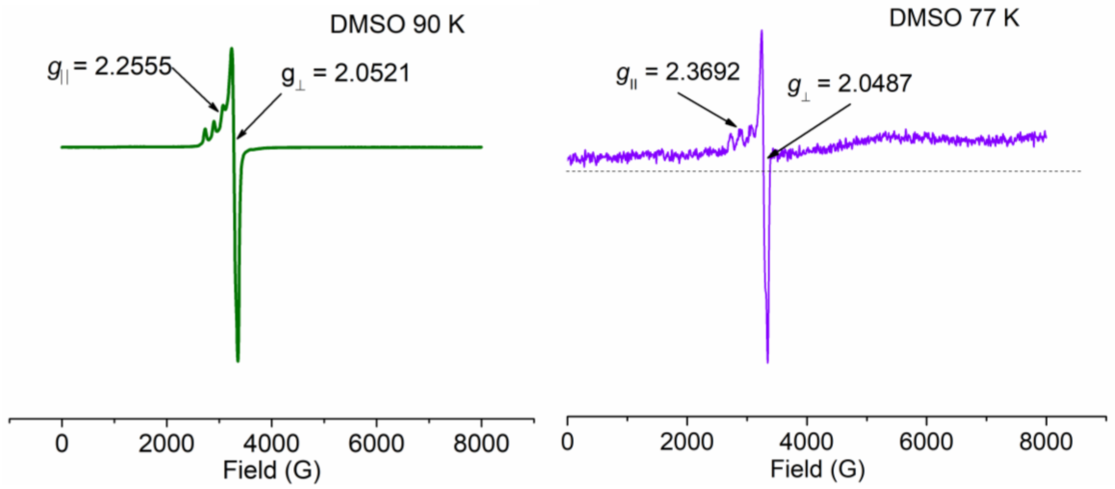

Figure S9*. ESR spectra measured in DMSO frozen solution of* ***2*** *(left) and* ***3*** *(right).*

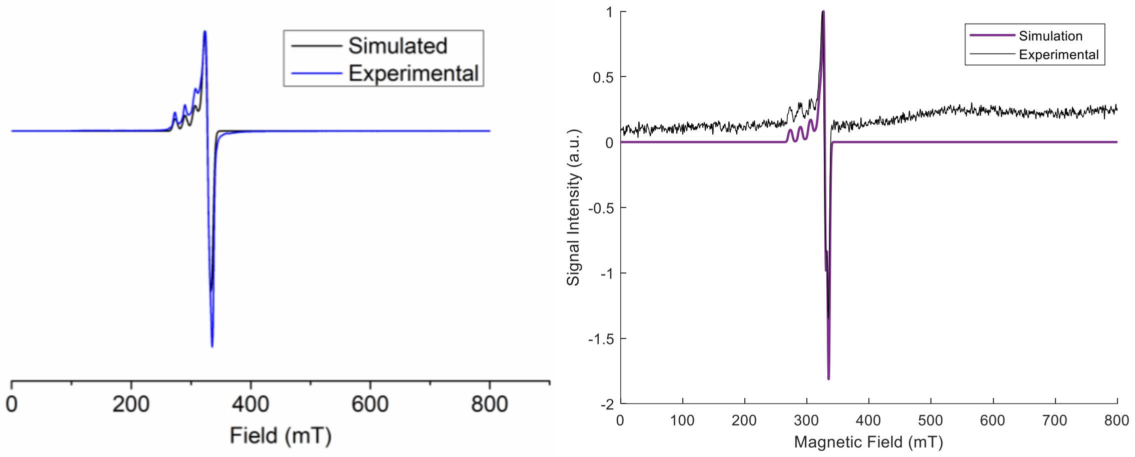

Figure S10*. ESR spectrum simulated and experimental in DMSO solution of* ***2*** *(left) and* ***3*** *(right).*
